# Analysis of the microRNA Expression Profile of Bovine Monocyte-derived Macrophages Infected with *Mycobacterium avium* subsp. *Paratuberculosis* Reveals that miR-150 Suppresses Cell Apoptosis by Targeting PDCD4

**DOI:** 10.3390/ijms20112708

**Published:** 2019-06-01

**Authors:** Zi Wang, Ling Cong Kong, Bo Yan Jia, Jing Rui Chen, Yang Dong, Xiu Yun Jiang, Hong Xia Ma

**Affiliations:** College of Animal Science and Technology, Jilin Agricultural University, Xincheng Street No.#2888, Changchun 130118, China; wangzi0225@jlau.edu.cn (Z.W.); lingcong@jlau.edu.cn (L.C.K.); jiaboyan@jlau.edu.cn (B.Y.J.); chenjingrui@jlau.edu.cn (J.R.C.); dongyang@jlau.edu.cn (Y.D.)

**Keywords:** *M. avium subsp. paratuberculosis*, microRNAs, high-throughput sequencing, miR-150, PDCD4, apoptosis

## Abstract

*M. avium subsp. paratuberculosis* (*MAP*) is the causative pathogen of Johne’s disease, a chronic granulomatous enteritis that principally affects ruminants and can survive, proliferate and disseminate in macrophages. MicroRNAs (miRNAs) are important regulators of gene expression and can impact the processes of cells. To investigate the role of miRNAs in monocyte-derived macrophages (MDMs) during *MAP* infection, we used high-throughput sequencing technology to analyze small RNA libraries of *MAP*-infected and control MDMs. The results showed that a total of 21 miRNAs were differentially expressed in MDMs after *MAP* infection, and 8864 target genes were predicted. A functional analysis showed that the target genes were mainly involved in the MAPK signaling pathway, Toll-like receptor signaling pathway, NF-kappa B signaling pathway and apoptosis. In addition, using a dual-luciferase reporter assay, flow cytometry, and a small interfering (si)RNA knockdown assay, the role of miR-150 in regulating macrophage apoptosis by targeting the programmed cell death protein-4 (PDCD4) was demonstrated. These results provide an experimental basis to reveal the regulatory mechanism of MAP infection and suggest the potential of miRNAs as biomarkers for the diagnosis of Johne’s disease in bovines.

## 1. Introduction

*Mycobacterium avium* complex (MAC) is an important zoonotic pathogen that causes respiratory tract, lymph node, and soft tissue infections in healthy individuals [1]. *Mycobacterium avium* (*M. avium*) and *Mycobacterium intracellulare* are the two main species belonging to the MAC. Moreover, secondary infection with *M. avium* has gained more attention due to immunocompromised individuals, such as AIDS patients [2,3]. *M. avium* is divided into four subspecies: *M. avium* subsp. *avium*, *Mycobacterium avium* subspecies *silvaticum*, *M. avium* subsp. *paratuberculosis* and *M. avium* subsp. *hominissuis* [4,5]. *MAP* is the causative pathogen of Johne’s disease, a chronic granulomatous enteritis that principally affects ruminants. The organism has a worldwide distribution, and the main transmission route of *MAP* is a fecal-oral route through milk, water and pasture [6,7]. The challenges and cost of the disease in the livestock industry have been increasing. In the United States, annual losses in the cattle industry have been estimated at between $200 million and $1.5 billion [8]. Furthermore, *MAP* may have a role in Crohn’s disease, a human chronic inflammatory bowel disease, although the causal association remains controversial [9,10]. In the infection of *MAP*, the bacilli are phagocytosed and impair host macrophage processes, resulting in subclinical infections. In addition, there is general consensus that MAP influences the apoptotic of bovine macrophages. At different stages of the infection, *MAP* may assume either pro- or anti- apoptotic roles [11]. For example, *MAP* initially postpones apoptosis to allow early intracellular replication, and later induce apoptosis to exit the cell when intracellular conditions no longer favor growth [12,13]. Analysis of the host macrophage mRNA and miRNA expression profile during infection can illuminate the molecular mechanisms and host-pathogen interactions associated with Johne’s disease. At present, the mRNA transcriptome of bovine monocyte-derived macrophage (MDM) response during *MAP* infection has been described [14,15].

MicroRNAs (miRNAs) are short, non-coding RNAs (19–24nt in length), which bind to the 3′ untranslated regions of target mRNAs to regulate the translation into protein or accelerate the decay of expressed transcripts [16]. Since their initial discovery, studies have demonstrated the roles of miRNAs in a wide range of cellular processes, such as cell proliferation, differentiation and apoptosis [17,18,19]. There are also some studies that demonstrated that miRNAs regulate innate and adaptive immune mechanisms [20,21]. Therefore, many studies have reported the roles of the miRNAs of host cell-pathogen interaction networks in humans and mice [22,23,24,25]. By contrast, studies of miRNAs in bovines are limited. Li Jizong et al. performed high-throughput sequencing to analyze small RNA libraries of CPIV3-infected and mock-infected MDBK cells, and 249 known and 152 novel miRNAs were differentially expressed in MDBK cells after CPIV3 infection [26]. Lewandowska-Sabat et al. identified the *Streptococcus agalactiae*-induced differential regulation of miRNAs in bovine macrophages, and the predicted target genes were significantly enriched in interleukin-4-mediated signaling events and the intrinsic pathway for apoptosis [27]. In the study of miRNA expression profile responses to *Streptococcus uberis* in vivo, Lawless et al. profiled the miRNA expression in both milk and blood monocytes, and 26 miRNAs and more than 3500 genes were identified as being significantly differentially expressed over 48 h. Pathway analysis revealed that the predicted target genes of down-regulated miRNAs were highly enriched in terms of innate immunity [28]. However, the miRNA expression profile of bovine monocyte-derived macrophages infected with *MAP* has not been reported. At present, the roles that miRNAs play in regulating immune responses and effects in response to infection are not too clear for bovines, compared to for humans and mice. Investigations into bovines were focused on characterizing the miRNA expression during bacterial or viral infections, but detailed mechanism research is lacking.

In this study, in order to gain a better understanding of *MAP* infection in immature macrophages (primary cells), high-throughput sequencing technology was used to perform an analysis of the miRNA profiles of bovine monocyte-derived macrophages, after *MAP* infection. Our study showed that the miRNAs play an important role in regulating mRNA during *MAP* infection, and furthermore, the identification of differentially expressed miRNAs may provide a basis for the development of biomarker assays for the early diagnostic of *MAP* subclinical infection. Moreover, the second part of our study characterized the role of miR-150 in regulating macrophage apoptosis by targeting PDCD4.

## 2. Results

### 2.1. Mapping and Annotation of miRNA Sequencing Data

The small-RNA libraries of *MAP*-infected and control MDM groups were sequenced. The total numbers of raw reads collected from the *MAP*-infected and control MDM cells were 14.3 and 17.0 million raw reads per library. After removing reads with a low quality and discarding sequences shorter than 18 nt and longer than 26 nt, 12.8 and 15.0 million clean reads were obtained, which corresponded to 89.1% and 88% of the raw reads, respectively. The number of raw reads per sample, the percentage of clean reads and the percentage of uniquely mapped reads for each sample are presented in Table S1. The length distribution of the clean reads was primarily 21–24 nt in length, and read counts of 22 nt were the most numerous (Figure S1). 

A total of 510 mature miRNAs were identified, among which a total of 433 miRNAs were homologous to bovines, while 77 were predicted to be novel miRNAs that are not homologous to any species (Table S2). The miRNA with the highest expression was bta-miR-21-5p, and bta-miR-16a, bta-miR-26b, bta-miR-142-5p, bta-miR-26a, bta-miR-223, bta-miR-16b, bta-miR-22-3p, bta-let-7g, bta-miR-146a, bta-let-7f, bta-miR-24-3p, bta-miR-191 were the top 13 most abundant miRNAs in all samples (Table S3).

### 2.2. Differentially Expressed miRNAs in MDM Challenged with MAP

**Figure 1:**
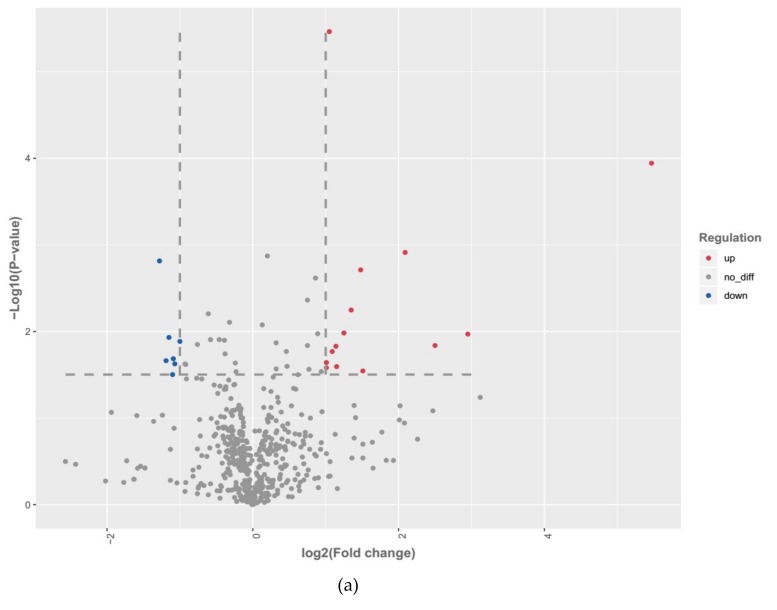

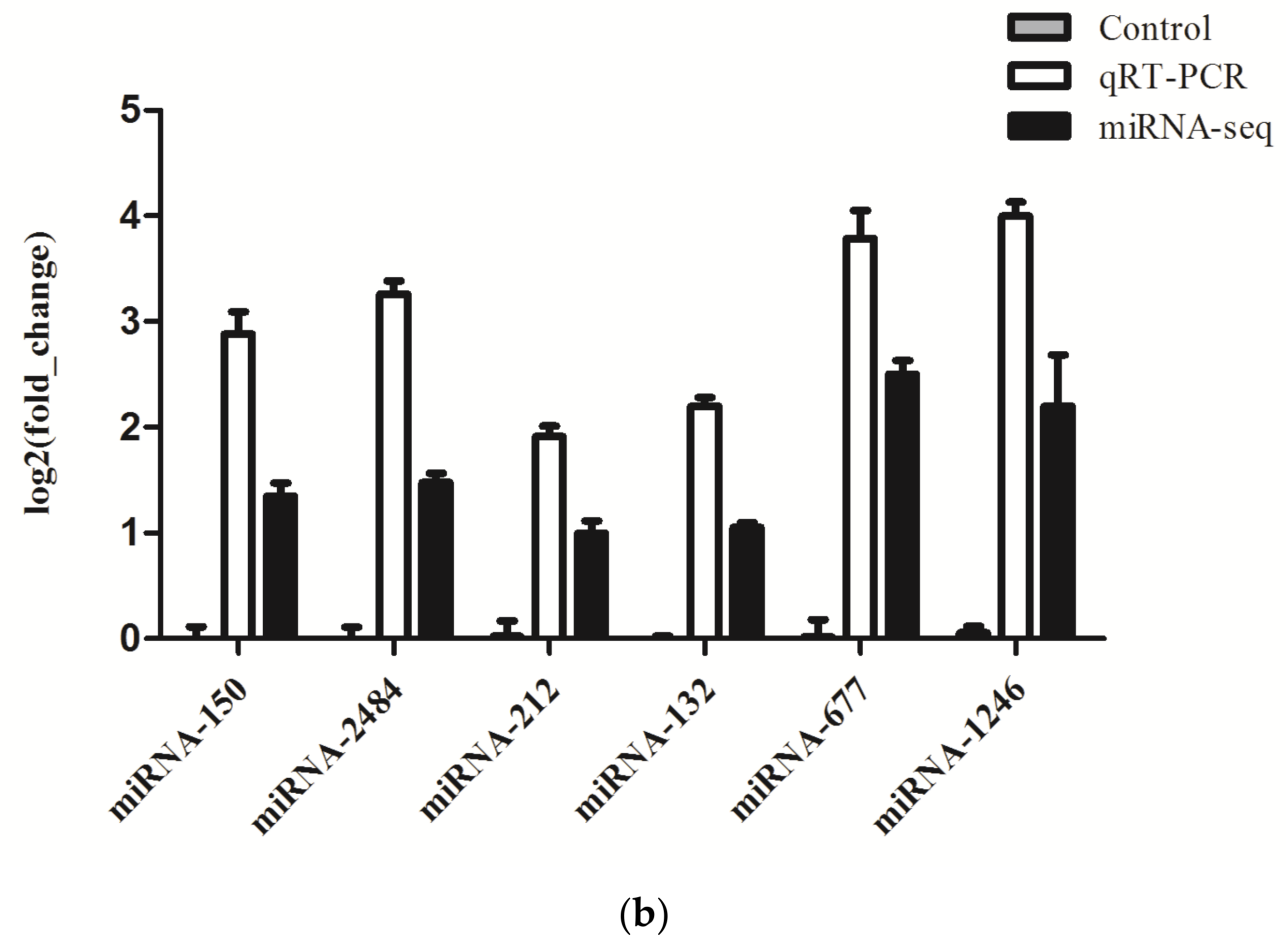
(**a**) Scatter plots showing the differentially expressed miRNAs in MDMs challenged with *MAP* compared to the control MDMs. Red, blue and gray are representative the upregulated, downregulated and unchanged miRNAs, respectively.; (**b**) Validation of the RT-qPCR analysis of bta-miR-677, bta-miR-132, bta-miR-1246, bta-miR-150, bta-miR-212 and bta-miR-2484.

The differential expression analysis revealed 21 differentially expressed miRNAs in MDM challenged with *MAP*, compared to the control MDM (detailed information is shown in Table S4). Among the differentially expressed miRNAs, 14 were up-regulated, and 7 were down-regulated (Figure 1a). 

To validate the results obtained from RNA-Seq, six miRNAs, including bta-miR-677, bta-miR-132, bta-miR-1246, bta-miR-150, bta-miR-212, bta-miR-2484, were selected to be examined by RT-qPCR. The results of RT-qPCR were in accordance with the sequencing data, which indicated that the results of our miRNA-seq were reliable (Figure 1b).

### 2.3. Prediction and Functional Characterization of Target Genes for differentially expressed miRNAs

**Figure 2:**
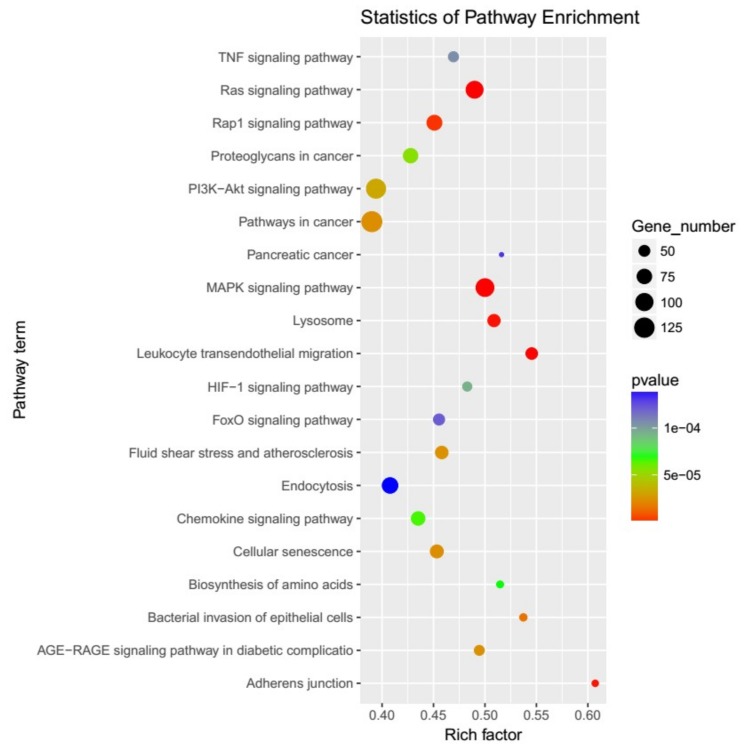
KEGG analysis of the predicted target genes, showing the top 20 prominent KEGG pathways.

To investigate the functions of the differentially expressed miRNAs, targetScan and miRanda were used to predict the mRNA targets. A total of 8864 genes were predicted to be potential miRNA targets (Table S5). Then, Gene Ontology (GO) and Kyoto Encyclopedia of Genes and Genomes (KEGG) functional enrichments were performed to analyze the potential biological functions of these target genes. The GO analysis of the target genes revealed that they were involved in the biological process, cellular component and molecular function (Table S6). The KEGG pathway mapping showed that the target genes were involved in many important biochemical, metabolic, and signal transduction pathways, such as the MAPK signaling pathway (ko04010), Ras signaling pathway (ko04014), apoptosis (ko04210), chemokine signaling pathway (ko04062) (Table S7). The 20 most prominent KEGG pathways are exhibited in Figure 2. 

### 2.4. Validation the Interaction of miR–150 and PDCD4

**Figure 3:**
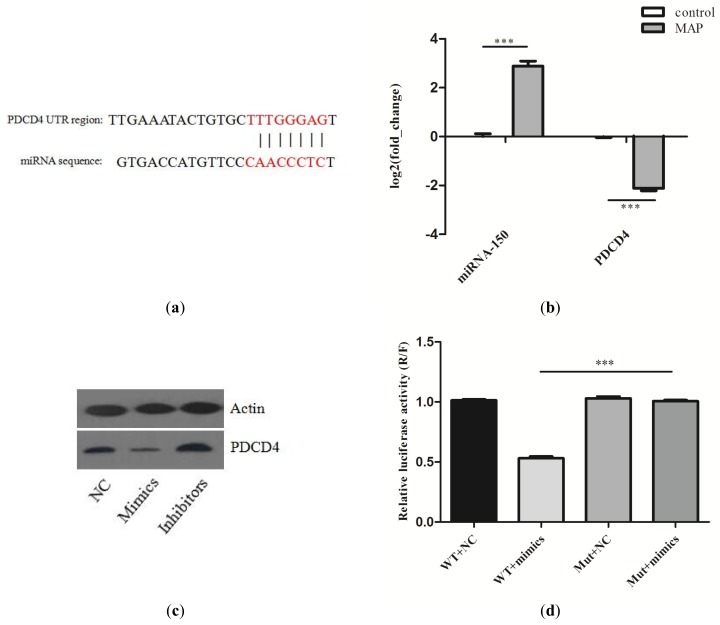
(**a**) Predicted binding sites of miR-150 in the 3′UTR region of the *PDCD4* sequence. The red labeled sequence represents the complementary region between miR-150 and the 3′ UTR of *PDCD4*; (**b**) RT-qPCR detected the expression changes of miR-150 and *PDCD4* during *MAP* infection; (**c**) Western blot analysis detected the expression of the PDCD4 in RAW264.7 cells transfected with NC, miR-150 mimics or miR-150 inhibitors; (**d**). Dual-luciferase reporter system. The H293 cells were co-transfected with miR-150 mimics and a luciferase reporter containing a fragment of the *PDCD4* 3’UTR that harbored either the miR-150 binding site (WT) or a mutant (MUT).

To study the function of differentially expressed miRNAs between *MAP*-infected and control MDM, the predicated bond between miR-150 and *PDCD4* was further studied (Figure 3a). The RT-qPCR results indicated that miR-150 and its target gene, *PDCD4*, had an inverse expression tendency in *MAP*-infected and control MDM (Figure 3b). The similarity analysis showed that miR-150 was conserved among bovines, mice and humans. Thus, we chose mouse RAW264.7 cells as the further experiment model. The expression of PDCD4 in RAW264.7 cells treated with miR-150 mimics or inhibitors was quantified using western blot. Compared with the negative control, the miR-150 mimics or inhibitors could suppress or increase the expression of PDCD4 (Figure 3c). The luciferase assay in HEK293 cells confirmed the inverse relationship between miR-150 and *PDCD4* (Figure 3d). miR-150 mimics that were significantly treated suppressed the luciferase activity of reporter genes containing the 3′UTR of *PDCD4*, while the mutated luciferase reporter was unaffected by miR-150 mimics overexpression of miR-150.

### 2.5. miR-150 Suppresses the Apoptosis of Macrophages by Targeting PDCD4

**Figure 4:**
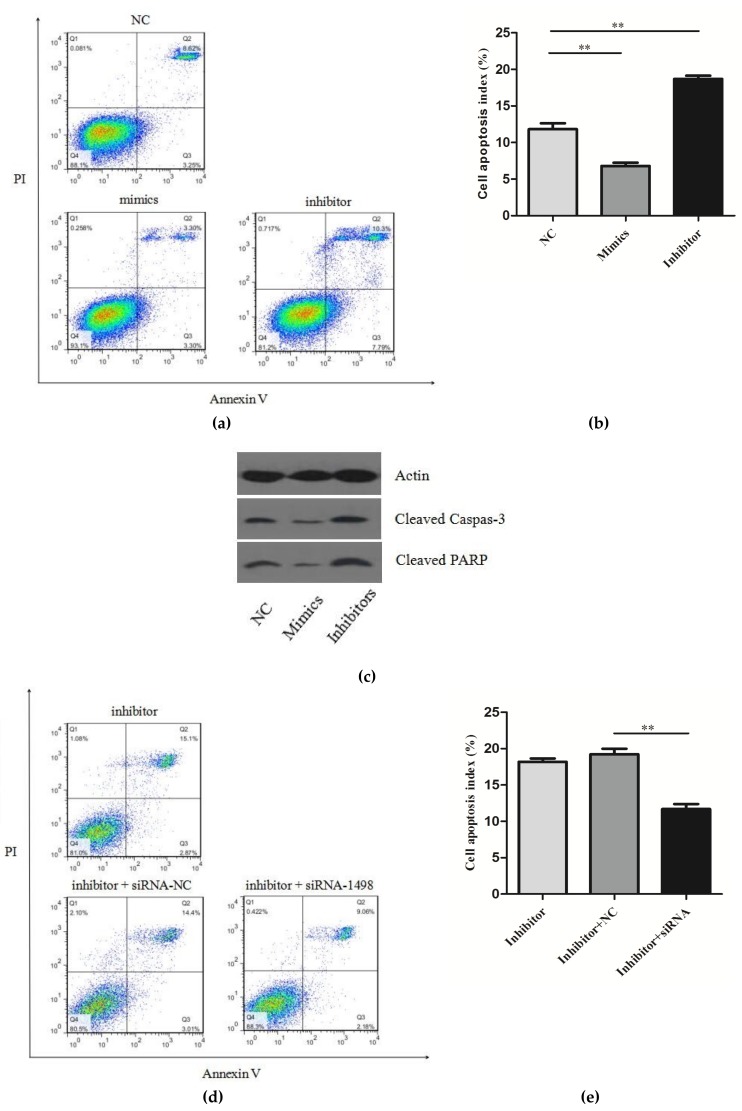
(**a**) Detection of the cell apoptosis of RAW264.7 transfected with NC, miR-150 mimics or miR-150 inhibitors; (**b**) Analysis results of the cell apoptosis index of the 3 groups in Figure 4a; (**c**) The expression of cleaved caspase-3 and cleaved PARP was detected by western blotting; (**d**) Detection of the cell apoptosis of RAW264.7 transfected with miR-150 inhibitors or RAW264.7 co-transfected with miR-150 inhibitors and siRNA; (**e**) Analysis results of the cell apoptosis index of the 3 groups in Figure 4d.

Next, we studied the role of miR-150 in regulating *PDCD4* in macrophage apoptosis. Firstly, using folw cytometry analysis, we investigated whether the overexpression of PDCD4 would affect cell apoptosis in RAW264.7 cells. To overexpress *PDCD4*, an expression plasmid, designed to specifically express the full-length ORF of *PDCD4*, without the miR-150 responsive 3′-UTR was constructed and transfected into RAW264.7 cells, and we found that the overexpression of PDCD4 significantly promoted cell apoptosis (Figure S2). Secondly, miR-150 mimics or inhibitors were transfected into the RAW264.7 cells. The Annexin V-FITC/PI staining assay showed that the apoptosis index in the group with miR-150 mimics was suppressed, and the group with miR-150 inhibitors was increased (Figure 4a,b). We also detected the expression of cleaved caspase-3 and cleaved PARP and found that it was significantly decreased in cells transfected with miR-150 mimics, whereas it was increased in cells transfected with miR-150 inhibitors (Figure 4c). Furthermore, an siRNA-targeting *PDCD4* was designed and simultaneously transfected with miR-150 inhibitors, and the siRNA dramatically rescued the increased effect of miR-150 on cell apoptosis (Figure 4d,e). These results reveal that *PDCD4* is crucial for the apoptosis of RAW264.7 cells and that miR-150 is able to suppress cell apoptosis by silencing *PDCD4*. 

## 3. Discussion

In recent years, high-throughput sequencing technologies have greatly enhanced the ability to understand the interaction between host macrophage and mycobacterial pathogens [29,30,31]. Johne’s disease in ruminants is caused by *MAP*, which is a mycobacterial species related to *M. tuberculosis* and *M. bovis*. Macrophages play an important role in the host-pathogen interaction of Johne’s disease. They can become the havens for survival, proliferation and dissemination of the MAP. At present, most studies using RNA-sequencing methods mainly focused on mRNAs, however, miRNAs have been revealed to be the regulatory factor of mRNA expression in inflammatory responses [32,33]. In this study, high-throughput sequencing was used to identify the cellular miRNA expression profile involved in the *MAP*-infected and uninfected MDMs. As previously reported, the majority of MDM transcriptional changes, induced by *MAP* infection, occur within the first 6 h [14]. Therefore, for our study, we analyzed the miRNA expression profile of MDMs at 6h post-infection. Based on the analysis of the clean reads, the majority of clean reads in MDMs had lengths of 21–24 nt, and reads of 22 nt were the most abundant in the sRNA libraries. These results were in accordance with the typical size of miRNAs, indicating that the libraries were mainly enriched with miRNA sequences.

In our study, 21 miRNAs were differentially expressed in *MAP*-infected MDMs, compared to uninfected control MDMs. Among these miRNAs, some of them, such as bta-miR-132, bta-miR-1246, bta-miR-150, and bta-miR-122, have been reported to have a positive or negative regulation during pathogen infection in bovines [34,35,36,37]. More recently, a study revealed that bta-miR-1246 was significantly up-regulated during the Caprine Parainfluenza Virus Type 3 (CPIV3) infection of MDBK Cells [26]. In addition, bta-miR-1246 showed the same expression trend in mammary glands and exosomes during Staphylococcus aureus infection [35,36]. In our study, the expression of bta-miR-1246 in MDMs infected with *MAP* was also significantly up-regulated, relative to the control MDMs (fold change: 4.25). These results indicate the possibility that bta-miR-1246 participates in host-pathogen interaction in different tissues and cells of bovines. However, there is a very limited number of functional studies on bta-miR-1246. Thus, more research on the function of the inflammatory response of bta-miR-1246 is recommended in the future. Furthermore, the differentially expressed bta-miR-132, bta-miR-2484, bta-miR-451, bta-miR-380-3p in our study were also detected in the report of the microRNA profiling of mammary glands infected with staphylococcus aureus [35]. miR-132 has been shown to play a role in controlling inflammatory responses. The miR-132 expression increased after virus infection and was reported to regulate the immune response by targeting p300, which demonstrated its role in the regulation of anti-viral immunity [38]. In another study, it was reported that the expression of miR-132 increased after the LPS-induced inflammation of rat alveolar macrophages, revealing that miR-132 may be involved in the regulation of macrophage inflammatory responses [39]. bta-miR-2484 is nearly species-specific, and it did not appear to be homologous in either humans or mice. In our study, the expression of bta-miR-2484 in MDMs infected with *MAP* was up-regulated, which associates it with mammary glands infected with Staphylococcus aureus. bta-miR-451 is a homolog to has-miR-451 and mmu-miR-451, and it has been reported to target MO25 in mouse heart tissue, altering the AMPK signaling [40]. miR-451 has also been reported to regulate the expression of cytokines in mice in response to influenza infection [41].

In our study, bta-miR-150 is another noteworthy differentially expressed miRNA. It is a homolog to has-miR-150-5p and mmu-miR-150-5p, and miR-150 was highly expressed in MDMs. miR-150 has been found in targeted-regulating MyD88 (a key regulator of TLR signaling) [42]. It has also been shown to target CXCR4 to regulate the mobilization and migration of bone marrow-derived mononuclear cells [43]. miR-150 also has been identified to regulate inflammatory responses in the macrophages of mice [44]. Therefore, a deeper analysis of the biological function and effect of the expression of miR-150 in MDMs needs to be clarified and it may participate in the regulation of *MAP* infection. Regarding the expression of bta-miR-122, our results showed that the expression of bta-miR-122 was significantly increased in MDMs infected with *MAP*. This result was consistent with another report on the expression profiling of peripheral blood miRNA in dairy cows with Escherichia coli-induced mastitis [37]. In addition, miR-122 also increases in the bloodstream in the murine liver injury model and has been predicted to be a biomarker of liver injury [45]. Therefore, we hypothesize that bta-miR-122 may be involved in the inflammatory response of *MAP* infection. Other differentially expressed miRNAs, bta-miR-92b, bta-miR-1343-3p and bta-miR-1306 were down-regulated in our study. A previous study observed that their down-regulation in bovine monocyte-derived macrophages being challenged with Streptococcus agalactiae [27]. The results suggest that bta-miR-92b, bta-miR-1343-3p and bta-miR-1306 may play a role in MDMs after bacterial infection and could be used as a candidate for further research.

It is noteworthy that bta-miR-677 and bta-miR-212 were moderately expressed and significantly increased in MDMs infected with *MAP*, and they have not been reported in previous studies of the pathogen infection of bovines. The early stage of *MAP* infection often remains asymptomatic with pathology largely restricted to the ileum, rendering diagnosis difficult [46]. The bta-miR-677 and bta-miR-212 may be used as potential diagnostics biomarkers of early *MAP* infection. However, due to the lack of infection control in our study, in future research, further investigation is needed to verify whether the present results are specific to viable *MAP* infection. 

In recent years, there have been several reports of miRNAs in the serum of *MAP*-infected cattle [47,48]. Some miRNAs, such as bta-miR-19b, bta-miR-301a, bta-miR-32, bta-miR-205, bta-miR-92b and bta-miR-432, were differentially expressed after *MAP* infection in serum. In our study, bta-miR-92b was also significantly differentially expressed in MDMs challenged with *MAP*. Moreover, bta-miR-19b showed the same expression trend in our results, but the results were not significant. Furthermore, there is a report on the alteration of the genomic expression profiles of long non-coding RNAs in bovine macrophages in response to *MAP* infection [49]. All of these studies advance our understanding of the roles of non-coding RNAs during *MAP* infection.

The biological analysis of miRNAs and the target genes in our study provided a basis for studying the immune regulation of *MAP* infection. In previous studies, the mRNA transcriptome of MDMs during *MAP* infection has been described, and in accordance with our study, the pathway analysis showed that the differentially expressed mRNAs were also involved in a number of important pathways, such as the MAPK signaling pathway, NF-kappa B signaling pathway and apoptosis. Among the differentially expressed miRNAs, bta-miR-12023 and bta-miR-1343-3p were mainly involved in regulating the MAPK signaling pathway, and 21 target genes were predicted to be involved in the pathway. bta-miR-214, bta-miR-133a and bta-miR-1246 were mainly involved in regulating the NF-kappa B signaling pathway. bta-miR-1246, bta-miR-1306, bta-miR-454 and bta-miR-150 were mainly involved in regulating the apoptosis pathway. However, further research is needed to confirm the predication results. The GO and KEGG analysis laid the foundation of further studies, which should involve building a miRNA—mRNA—protein regulation network.

In the previous report, it was demonstrated that populations of *MAP*-infected MDMs contain fewer apoptotic cells than similar populations of control cells, and *MAP*-infected cells contain reduced caspase activity for caspases 3, 8, and 9 [12]. In this study, we further investigated the role of miR-150 in regulating apoptosis by targeting *PDCD4*. However, cell apoptosis is a complicated polygene-related process, meaning more research is needed to further clarify the regulatory mechanism of apoptosis. The *PDCD4* gene was first identified in the process of apoptosis, and it is generally thought that *PDCD4* participates in tumorigenesis through the regulation of apoptosis [50,51]. However, *PDCD4* is ubiquitously expressed in normal tissue, and the up-regulation of the PDCD4 protein has been identified in apoptotic cells [52,53]. In addition, *PDCD4* has also been reported to play an important role in various inflammatory diseases [54,55]. A recent study reported that miRNA-150 inhibitors could enhance the cell apoptosis of melanoma by targeting *PDCD4* [56]. In our study, miR-150 was highly expressed in MDMs and was significantly up-regulated after *MAP* infection. Conversely, its target gene *PDCD4* was down-regulated, as detected by qRT-PCR. In our studies, we showed that miR-150 could affect cell apoptosis and that miR-150 inhibitor-induced cell apoptosis was reversed by silencing *PDCD4* through siRNA. These results suggest that *PDCD4* is a mediator of macrophage apoptosis regulation by miR-150 in RAW264.7 cells. However, the exact mechanisms by which *PDCD4* induces apoptosis need to be elucidated in further studies. Our study partially confirmed the correlation between miRNA and mRNA, and the regulatory relationships of other miRNA–mRNA pairs still require further validation.

## 4. Materials and Methods 

### 4.1. Ethics Statement

This study was carried out in accordance with the recommendations of the ’Laboratory animal-guidelines for the ethical review of animal welfare (GB/T 35892-2018), General Administration of Quality Supervision, Inspection and Quarantine of the People’s Republic of China’. The protocol was approved by the AnimalCare and Use Committee of the Jilin Agricultural University (GB/T 35892-2018, approval at 6 February in 2018) and the owner of the cattle agreed to the experiment. 

### 4.2. Purification of Bovine Monocyte Derived Macrophages

The experimental method used in our study was previously described [14,15]. Briefly, six healthy Holstein cows aged 3–6 years were used in the current study. All animals were maintained under uniform housing conditions and nutritional regimens at the Ground Dairy Industry (Ground Dairy Industry Ltd, Changchun, China) farm. The animals did not have a recent history of Johne’s disease, and the cows were tested negative for paratuberculosis, as determined by the culture of fecal samples and a PCR assay of the conserved IS element (IS900) of fecal samples. For the monocyte isolation, 200 mL of whole blood was collected from the neck in sterile glass bottles with sodium citrate. Peripheral blood mononuclear cells (PBMC) were extracted by density gradient centrifugation on a Histopaque® 1077 (Sigma-Aldrich, Shanghai, China). Contaminated red blood cells (RBC) were removed following resuspension and subsequent incubation of the PBMC in RBC lysis buffer (Sangon Biotech, Shanghai, China) for 5 min at room temperature. Monocytes were then isolated using the anti-human CD14 MACS MicroBeads (Miltenyi Biotec Ltd., Shanghai, China), according to the manufacturer’s instructions and as previously described [15]. The purity of selected cells was verified by flow cytometry using an anti-CD14 fluorescein-labeled antibody, and they yielded a purity of CD14+ cells ≥ 95% (Figure S3). The CD14+ cells were subsequently grown in 24-well dishes at a density of 1 × 10^6^ cells per well in an RPMI 1640 medium supplemented with 10% FCS (Invitrogen, Shanghai, China), 1% non-essential amino acids and gentamicin (50 μg/mL; Sangon Biotech, Shanghai, China). Cells were left overnight at 37 °C in an atmosphere with 5% CO_2_, and were allowed to differentiate into an early-stage adherent macrophage phenotype that was confirmed by microscopy.

### 4.3. Bacterial Infection

The *MAP*-10 strain was received at the China Institute of Veterinary Drugs Control and cultured in Middlebrook 7H9 supplemented with 2 mg/ml Mycobactin J and a 10% Middlebrook OADC enrichment (BD Biosciences, Shanghai, China). The *MAP* cultures were grown at 37 °C for at least 8 weeks, until the bacteria were harvested for infections, and the growth was measured at an optical density (OD) of 600 nm. The cultures were frozen in 20% glycerol stocks at −80 °C, and the colony counts were determined as previously described [57]. For the infection, MDM cells were assigned to two groups (each group contains three replications): one group was infected with MAP, and the other group was uninfected. All cell cultures were infected, at a multiplicity of infection of 5:1. After 2h, post-infection, the media was replaced with fresh culture media containing gentamicin to prevent the growth of remaining extracellular bacteria and re-incubated at 37 °C and 5% CO_2_. At 6 h post-infection, the cells were harvested, and the infected and non-infected control MDMs were lysed and stored at −80°C until required for RNA extraction.

### 4.4. Library Preparation, Sequencing and Data Analysis

The total RNA was extracted using a Trizol reagent (Invitrogen, Carlsbad, CA, USA), referring to the manufacturer’s procedure. The total RNA quantity and purity were analyzed by a of Bioanalyzer 2100 (Agilent Technologies, Santa Clara, CA, USA), with RIN numbers at least 9.2 (MAP-1: 9.6; MAP-2: 9.7; MAP-3: 9.6; C-1: 9.7; C-2: 9.7; C-3: 9.2). About 1 µg of the total RNA was used to build a small RNA library, according to the protocol of TruSeq Small RNA Sample Prep Kits (Illumina, San Diego, CA, USA). Then single-end sequencing (50 bp) was performed on an Illumina Hiseq 2500 at the LC-BIO (Hangzhou, China), following the recommended protocol.

The raw reads were processed, using an ACGT101-miR (LC Sciences, Houston, TX, USA) to remove adapter dimers, junk, low complexities, repeats and common RNA families (rRNA, tRNA, snRNA, snoRNA). Subsequently, unique sequences, with a length of 18~26 nt, were mapped to Bos taurus in miRBase 21.0 by a BLAST search to identify the known miRNAs and novel miRNAs. Sequences containing varied lengths at both 3’ and 5’ ends and single mismatch within the sequence were retained in the alignment. The unique sequences, mapping to specific species’ mature miRNAs in hairpin arms were identified as known miRNAs. The unmapped sequences and the hairpin RNA structures containing sequences were predicted using the RNAfold software (University of Vienna, Vienna, Austria) (http://rna.tbi.univie.ac.at/cgi-bin/RNAfold.cgi).

The raw data from the miRNA sequencing were submitted to the GEO of NCBI, with accession numbers GSM3722592, GSM3722593, GSM3722594, GSM3722595, GSM3722596 and GSM3722597, for the miRNA data of the MAP-infected and control groups, respectively.

### 4.5. Differential Expression Analysis, Target Prediction and Functional Analysis

In order to compare the differential miRNA expression of MAP-infected and control MDMs, raw miRNA counts from each sample were used to normalize the expressions of miRNAs [58]. The miRNAs with fold-change in expression log2 (*MAP*-infected /control) ≥ 1 and *p*-value < 0.05 were considered as significantly upregulated or downregulated.

To predict the mRNA targets by differentially expressed miRNAs, two algorithms of target prediction were used to identify the binding sites (TargetScan 50 and Miranda 3.3a). Finally, the overlaps predicted by both algorithms were calculated. The GO analysis and KEGG pathway enrichment of these mRNA targets9 were performed using Blast2GO (version 2.8, which can be accessed online: http://www.blast2go.com) and KAAS (KEGG Automatic Annotation Server, which can be accessed online: https://www.genome.jp/kegg/kaas/) for Annotation.

### 4.6. Real-time Quantitative PCR (RT-qPCR)

The method of real-time quantitative PCR was used to validate the expression of 6 differentially expressed miRNAs and *PDCD4* genes. The total RNA was converted to cDNA using the PrimeScript RT regent Kit (TaKaRa, Dalian, China). Random primers, oligo dT or miRNA-specific stem-loop primers were designed by us and used for the reverse-transcribed cDNA. RT-qPCR was performed using the SYBR Green PCR Master Mix Reagent Kit (TaKaRa, Japan), U6 was used for the normalization of miRNA data and β-actin was used for the normalization of the mRNA data. The RT-qPCR procedure was as follows: 95 °C for 15 s, 60 °C for 1 min, and 95 °C for 15 s. The fold-change of the expression of the transcript mRNA or miRNA were analyzed using the 2^−ΔΔCt^ method. The RT-qPCR primers are listed in Table S8.

### 4.7. Cell Culture

HEK293 cells and RAW264.7 macrophages were cultured in high-glucose Dulbecco’s modified Eagle’s medium (DMEM; Hyclone, Carlsbad, CA, USA) supplemented with 10% fetal bovine serum (FBS; Hyclone, Carlsbad, CA, USA) at 37 °C with 5% CO_2_.

### 4.8. Vector Construction

The miR-150 mimics and inhibitors, and the negative control (NC), were compounded by GenePharma (Shanghai, China). The *PDCD4*-3’UTR, including the miRNA binding site and its mutation were cloned into the psi-CHECK2 dual-luciferase reporter vector (Promega, USA) named psi-CHECK2-PDCD4 (wild type, WT-PDCD4) and psi-CHECK2-PDCD4-mut (Mut-*PDCD4*). The overexpression vector pcDNA3.0-*PDCD4* was obtained by PCR amplification from the genomic DNA, using primers (Table S1) containing restriction enzyme sites BamHI/EcoRI (TaKaRa, Japan). The sequence information is listed in Table S8.

### 4.9. Cell Transfection

To detect the apoptosis-promoting effect of *PDCD4*, pcDNA3.0-PDCD4 was transfected into RAW264.7 cells. The cells at 90% confluence were plated in 6-well plates. When the growth reached approximately 80% confluence, 2 μg of pcDNA3.0-PDCD4 was transfected into the RAW264.7 cells using Lipofectamine 2000 (Invitrogen, USA).

### 4.10. Luciferase Activity Assay

HEK-293 cells were cultured in 24-well plates when the cell growth reached about 80% confluence. The miR-150 mimics and WT-PDCD4 or Mut-PDCD4 were co-transfected into cells by Lipofectamine2000. The cells were lysed at 24 h after transfection, and the luciferase activity in the cell lysates was measured using a dual-luciferase reporter assay system (Vazyme Biotech co., ltd, Nanjing, China), according to the manufacturer’s instructions.

### 4.11. Cell Apoptosis Assay

To detect the effect of miR-150 on macrophages apoptosis, miR-150 mimics and inhibitors and NC, were transfected into RAW264.7 cells respective. The siRNA of *PDCD4* (siPDCD4) and miR-150 inhibitor were transfected into RAW264.7 cells to confirm the miR-150 effect on cell apoptosis. The sequence of siRNA used in the transfection and NC siRNA are listed in Table S7.

Cell apoptosis was measured by Annexin V-FITC/PI staining assays. RAW264.7 cells were cultured in 6-well or 24-well plates. When the cells reached a confluence of 80–90%, the cells were transfected with miR-150 mimics and inhibitors, pcDNA3.0-PDCD4 or siRNA. After 24 h of incubation, the RAW264.7 cells were harvested in a 1.5 mL centrifuge tube and washed with a PBS buffer, resuspending them in a 500 μL 1× binding buffer. Cells were then treated with an Annexin V-FITC/PI Apoptosis Detection Kit (Vazyme Biotech co., ltd), and incubated in the dark for 10 min at room temperature, and then the cell apoptosis was immediately analyzed using a Flow Cytometer and the quantitative analysis of the apoptotic cell ratio was performed.

### 4.12. Western Blotting

The total proteins were extracted using a protein Lysis buffer RIPA containing 1 mM PMSF (Solarbio; Beijing, China). The protein levels were normalized by probing the same blots with a β-actin antibody. The extracts were boiled with an SDS loading buffer and separated by SDS-PAGE. The membrane was incubated overnight at 4 °C with primary antibodies specific to anti-PDCD4 (CoWin Biosciences, Beijing, China), anti-β-actin (CoWin Biosciences, Beijing, China), anti-cleaved-CASP3 (Cell Signaling Technology Inc., Shanghai, China), anti-cleaved PARP (Cell Signaling Technology Inc., USA). Anti-immune rabbit IgG-HRP (Sungene Biotech, Tianjin, China) served as a secondary antibody. The intensity of each band was scanned and quantified using the image J software.

### 4.13. Statistical Analysis

All data are expressed as the mean ± standard error based on 3 independent experiments. To identify the significant differences, comparisons were analyzed by a one-way analysis of variance for the P-value using GraphPad Prism 5 (GraphPad Software, San Diego, CA, USA). A *p*-value < 0.05 was considered statistically significant.

## 5. Conclusions

In summary, using high-throughput sequencing, the miRNA expression patterns in MDM responses to *MAP* infection were identified. A total of 467 known bovine miRNAs and 78 novel candidate miRNAs were detected. Subsequently, 21 differentially expressed miRNAs were identified from the two groups. A target prediction and functional analysis of these miRNAs suggested that they may play an important role in regulating the host immune response after *MAP* infection. We characterized and functionally evaluated the role played by one of the differentially expressed miRNA, miR-150. The results of several independent experiments suggest that miR-150 regulates macrophage apoptosis by targeting *PDCD4*. This is the first report on the miRNA expression profile in *MAP*-infected MDMs and may provide a basis for revealing the regulatory mechanism of *MAP* infection and the potential role of miRNAs as biomarkers in early diagnosis.

## Supplementary Materials

Supplementary materials can be found at https://www.mdpi.com/1422-0067/20/11/2708/s1.

## Abbreviations

*MAP*	*M. avium* subsp. *paratuberculosis*
miRNAs	MicroRNAs
MDMs	Monocyte-derived macrophages
*M. avium*	*Mycobacterium avium*
GO	Gene Ontology
KEGG	Kyoto Encyclopedia of Genes and Genomes
PDCD4	programmed cell death protein-4
