# Supplementary figures and images for: Analysis of the microRNA Expression Profile of Bovine Monocyte-derived Macrophages Infected with Mycobacterium avium subsp. Paratuberculosis Reveals that miR-150 Suppresses Cell Apoptosis by Targeting PDCD4
Source: Int J Mol Sci. 2019 Jun 1;20(11):2708. doi: 10.3390/ijms20112708 (PMC6600136; doi:10.3390/ijms20112708)

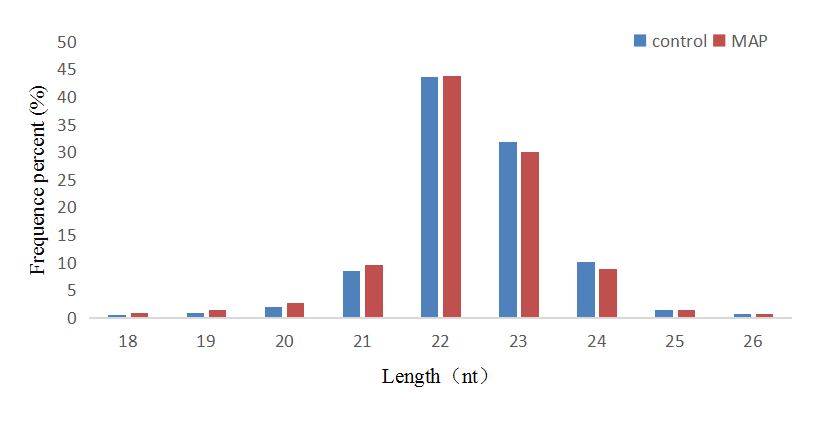

Supplement: Supplementary file 1 [file ijms-20-02708-s001.zip › Supplementary Materials/Figure S1. Length distribution of the clean reads of the sequences.tif]

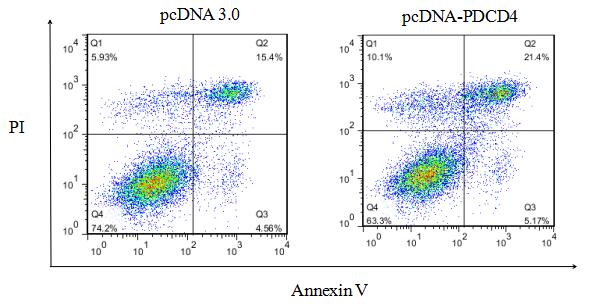

Supplement: Supplementary file 1 [file ijms-20-02708-s001.zip › Supplementary Materials/Figure S2a. Detection of cell apoptosis of RAW264.7 transfected with overexpression vector pcDNA3.0-PDCD4.jpg]

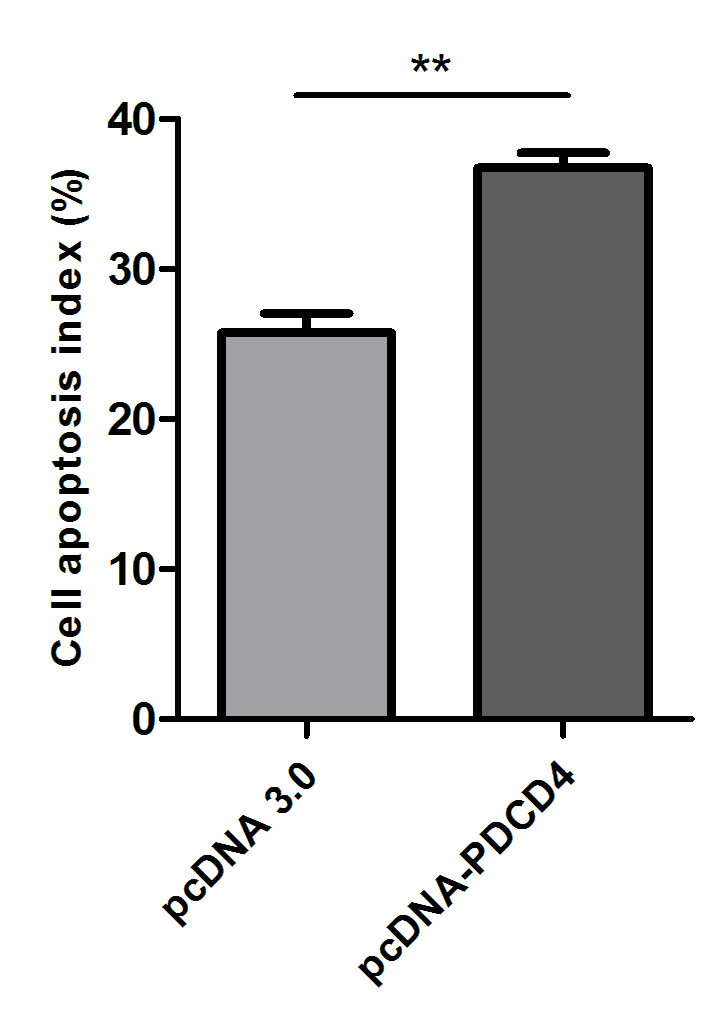

Supplement: Supplementary file 1 [file ijms-20-02708-s001.zip › Supplementary Materials/Figure S2b. Analysis results of cell apoptosis index of figure S2a.tif]

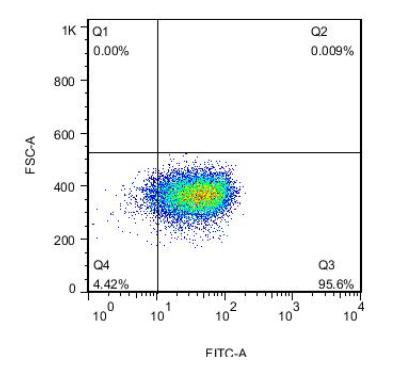

Supplement: Supplementary file 1 [file ijms-20-02708-s001.zip › Supplementary Materials/Figure S3. The result of purity of CD14+ cells.jpg]
