# Supplementary material for: Analysis of the microRNA Expression Profile of Bovine Monocyte-derived Macrophages Infected with Mycobacterium avium subsp. Paratuberculosis Reveals that miR-150 Suppresses Cell Apoptosis by Targeting PDCD4
Source: Int J Mol Sci. 2019 Jun 1;20(11):2708. doi: 10.3390/ijms20112708 (PMC6600136; doi:10.3390/ijms20112708)
Supplement: Supplementary file 1 [file ijms-20-02708-s001.zip › Supplementary Materials/Figure caption of the supplementary figures.doc]

Figure S1. Length distribution of the clean reads of the sequences. The x-axis indicates the length of reads. The y-axis indicates the percentage of each length.

Figure S2. (a) Detection of the cell apoptosis of RAW264.7 transfected with overexpression vector pcDNA3.0-PDCD4; (b) Analysis results of the cell apoptosis index of the 3 groups in Figure S2a.

Figure S3. The result of purity of CD14+ cells.
